# Organic or psychological? It does matter!

**DOI:** 10.1590/S1677-5538.IBJU.2022.99.11

**Published:** 2022-01-28

**Authors:** Flavia Ramos Glina, Sidney Glina

**Affiliations:** 1 Universidade do Porto Faculdade de Psicologia e Ciências da Educação Porto Portugal Faculdade de Psicologia e Ciências da Educação da Universidade do Porto, Porto, Portugal; 2 Disciplina de Urologia do Centro Universitário - FMABC Santo André SP Brasil Disciplina de Urologia do Centro Universitário - FMABC - Santo André, SP, Brasil

## COMMENT

Sexology formally began as a science in 1907 when Iwan Bloch proposed the creation of a scientific area for understanding sex and named it Sexualwissenschaft (sexual science or sexology), in German. In 1908 Magnus Hirschfeld published the first magazine dedicated to sexual science; (Zeitschrift Fur Sexualwissenschaft); in 1913 the first sexology society was founded - (the Medical Society for Sexology and Eugenics) by Magnus Hirschfeld, Iwan Bloch, Albert Eulenburg and in 1921 the first sexology congress was held (1).

Until the 20th century, it was believed that the basis of sexual dysfunctions was almost entirely psychological, mainly due to little knowledge of the human sexual response physiology. According to Masters and Johnson, in 1970, 90% of sexual impotence cases were psychogenic (2).

Human sexuality is based on perfect psyche-body functioning and dysfunctions usually result from an imbalance in this binomial. The evolution of knowledge on male and female physiology sexual responses made it possible to understand that various organic processes could also lead to erectile disorders (3). Many authors currently consider that most erectile dysfunction (ED) causes are organic (4).

This new knowledge quickly led to the medicalization of the treatment of sexual dysfunctions, reinforced by the launch of phosphodiesterase type 5 inhibitors (PDE5 i). They provide a safe and effective way to improve erectile response and also give the patient and physician the feeling that sexual dysfunctions can be solved with medications.

Although these drugs can be of great help to patients with psychogenic ED, the fact of using a drug to treat a sexual dysfunction has, in turn, led to an “organicization” of sexual dysfunctions. Everything started to have an “organic” cause.

However, on what is this “medical reductionism” as defined by Janinni et al. (4) based? Risk factors started to be confused with etiology. Diabetic men are at higher risk of developing ED, however, diabetes is not the cause, but diabetic neuropathy, which affects some diabetic men. The same occurs with other risk factors such as obesity, smoking, metabolic syndrome, etc. When a diabetic or hypertensive patient complains of ED, the doctor immediately thinks of organic ED, without being interested in an emotional cause that may be over-combined or prevalent. And immediately prescribes a pill of a PDE-5i. Sometimes, when patient does not get a good response for any reason or is not interested on use the medication, he can receive an indication of penile implant.

On the other hand, the workup for diagnosing the organic causes of ED is very imprecise. The diagnosis usually needs to be made through anamnesis and from the perspective of the investigator. Most of the complementary exams used are inaccurate and very prone to errors, which facilitates the lack of interest in the search for the etiology of ED.

The best propaedeutic tool to differentiate between psychogenic and organic ED is nocturnal penile tumescence tests. Although some question it (5), the presence of a rigid erection during sleep proves that the nerve pathways, penile arterial irrigation, and the veno-occlusive system function properly (6). However, tests in sleep laboratories are very expensive, inaccessible and many laboratories are not equipped to measure nocturnal penile tumescence. Devices that assess nocturnal tumescence at home (eg Rigiscan™) have practically disappeared from the market due to lack of demand.

Another widely used test is the penile artery Doppler ultrasound for diagnosing penile vascular dysfunctions. This test depends on the relaxation of cavernous sinusoidal smooth muscle by vasoactive drugs injected into the penis. However, this test is influenced by the patient's state of anxiety, which negatively impacts smooth muscle relaxation (7). Additionally, frequently more than one injection is needed (8), the action of drugs can be impacted by smoking (9) and this test is very prone to false-positive results (10). The same considerations apply to the different types of cavernosometry that have been described over the years; all depend on the action of vasoactive drugs on the smooth muscles of the corpora cavernosa and are greatly influenced by the individual's adrenergic state (11).

When thinking about possible neurological etiologies such as diabetic or alcoholic neuropathies, or even damage to neurovascular bundles during radical prostatectomy, we must remember that there is no electroneuromyographic method that assesses penile autonomic innervation. According to Giuliano and Rowland, no neurophysiological exam is capable of evaluating the integrity of the pro-erectile penile innervation and should not be recommended for the evaluation of patients with ED (12). In the past, electromyography of the corpora cavernosa has been tried, but unfortunately, the results have not advanced and this method is still considered experimental (12).

The development and subsequent release of PDE-5i reinforced the “ED organicity”. They have been widely studied in multicenter, randomized, placebo-controlled investigations in thousands of patients. Hatzimouratidis (13) wrote a review of sildenafil studies and reported high effectiveness in different groups of patients with ED. Except for patients with ED after radical prostatectomy, which can be considered a cause of ED, the other groups were composed of patients with risk factors: hypertensive, coronary artery disease, diabetics, etc. Every investigator who took part in these studies (14) remember that the cause of ED, whether organic or psychological, was at the investigator's discretion in most of the studies and no exam was performed to determine it.

On the other hand, the proper response of PDE-5i depends on an integral organic substrate. These drugs promote an active inhibition of the PDE-5 enzyme increasing cyclic guanosine monophosphate (cGMP), which facilitates smooth muscle relaxation (15). However, to occur an erection there must be the arrival of pro-erectile stimulation to the penis, production of neuronal nitric oxide, production of cyclic GMP, relaxation of the smooth muscles of the corpus cavernosum, increase in penile arterial flow and occlusion of the venous system. Men with substantial alteration in this system, whether due to neuropathy, occlusion of the arteries, or fibrosis that prevents veno-occlusion, do not get an adequate erectile response with these medications. It can be at least suggested that most patients who respond well to PDE-5 inhibitors have a conserved erection mechanism and may not have an organic cause for their ED.

But what does all this matter? We have adequate treatments, many of them minimally invasive, safe, and affordable. Why is it important to know whether ED is psychogenic or organic?

The use of PDE-5i in patients with psychogenic ED can take away the opportunity of treating the dysfunction with psychological therapy. Additionally, many individuals do not use the medication as they should after some time. An example is hypertensive or diabetic patients who end up abandoning treatment or not using the appropriate doses.

Non-definition of the cause of ED and its adequate treatment may be the reason for the high discontinuation rate and patient dissatisfaction that the use of PDE-5i presents. Corona et al. (16) showed that, despite being highly effective, almost 50% of patients give up on its use during the first year of treatment. They identified that problems related to partnership and lack of effectiveness are mainly responsible for these rates. At the very least, problems with the partnership could be identified and addressed by psychological intervention. Atallah et al., in a recent published systematic review, reported that the combination of psychological interventions and the treatment with PDE-5 inhibitors are more effective on restoring erectile function and sexual satisfaction in patients with erectile dysfunction when compared with any of the treatments alone (17).

Failure to identify symptoms such as anxiety and depression, which are recognized causes of ED (4), can make the patient's treatment more difficult and lead to inappropriate conduct, as well as ineffective results.

Dos Reis et al. showed that 27.6% of patients who would undergo penile prosthesis implantation for the treatment of ED and who were evaluated by a psychologist in the preoperative period presented symptoms of anxiety and depression that led to the suspension of the surgery (18). Along the same lines, Trost et al. (19) identified what they called CURSED (compulsive/obsessive, unrealistic, revision, surgeon shopping, entitled, denial, and psychiatric patients) who are patients at a high risk of dissatisfaction after penile prosthesis implantation. On many occasions, these are not easily identifiable patients, and the risks of poor selection range from postoperative complications and patient dissatisfaction to legal proceedings, loss of credibility, and harm to the patient (20).

In our practice, we seek to assess the possible causes of ED and in the same way that we make sure to check testosterone levels to assess hormonal homeostasis, or a Doppler ultrasound to assess penile circulation, we also ask for an assessment with a psychologist/sexologist to evaluate the emotional status of the patient. Recently, the European Society of Sexual Medicine (ESSM) stated that a proper assessment of ED requires a medical and psychosocial evaluation and a multidisciplinary treatment has proven to be more effective in treating ED (21). While medications are focused on treating the symptoms, psychological treatment might help by promoting treatment adherence, as well as addressing psychological correlates and it can help on preventing a recurrence of the sexual dysfunction since patients learn to manage their dysfunctional response patterns (22).

It is important to remember that ED is multifactorial and the patient needs to be fully evaluated before starting any treatment. More and more, we see patients who use PDE-5i medications successfully but also have a desire for a more definitive solution, which usually can involve the resolution of emotional conditions.
